# In Vivo Electrochemical Monitoring of Safinamide Pharmacokinetics in the Brain Explores Its Correlation With Vision‐Related Neuronal Activity

**DOI:** 10.1002/advs.75618

**Published:** 2026-05-10

**Authors:** Xiaoke Nan, Chuqi Li, Qianhe Dai, Junlan Zhou, Lijiao Cao, Yuying Liu, Jinger Chen, Meiping Xiong, Yuhang Jiang, Xianchan Li

**Affiliations:** ^1^ State Key Laboratory of Natural and Biomimetic Drugs School of Pharmaceutical Sciences Beijing Key Laboratory of Carbohydrate Intelligent Manufacture and Functional Applications Peking University Beijing P. R. China

**Keywords:** electroanalysis, in vivo, neuronal activity, pharmacokinetics, safinamide

## Abstract

Understanding drug pharmacokinetics (PK) at its site of action, particularly in the brain, is essential for accurately evaluating therapeutic efficacy and safety. However, conventional PK assessment based on blood measurements often fails to reflect drug dynamics in the central nervous system (CNS). Here, taking the anti‐Parkinson's drug safinamide (SAF) as an example, we introduce a real‐time in vivo electrochemical sensing strategy with high spatiotemporal resolution, operational simplicity, and excellent reproducibility. Our results reveal that the PK profile of SAF in the substantia nigra (SN) differs markedly from that in plasma, characterized by enhanced brain accumulation and distinct clearance kinetics. By simultaneously monitoring SAF and neuronal activity in the superficial gray layer (SuG) of the superior colliculus (SC), we observed pronounced suppression of neuronal firing at peak SAF levels, followed by gradual recovery during SAF elimination. This tight temporal correlation suggests that transient inhibition of vision‐related neuronal activity may be directly associated with SAF concentration at the site. Overall, our study establishes an electrochemical approach enabling rapid, selective, and real‐time drug monitoring in the living brain, offering a powerful tool to promote mechanistic studies of CNS drug action and safety.

## Graphical abstract

An electrochemical platform based on differential pulse amperometry enables real‐time, selective tracking of safinamide pharmacokinetics in the living brain with high spatiotemporal resolution. When integrated with electrophysiology, this approach reveals that drug‐induced suppression of vision‐related neuronal activity provides mechanistic insight into safinamide‐associated retinal effects, offering a broadly applicable strategy for mechanistic neuropharmacology.

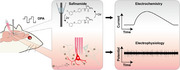

## 1 Introduction

Determination of a drug's effective concentration at its site of action requires monitoring of its spatial and temporal distribution in living organisms, which is strongly influenced by complex and heterogeneous tissue microenvironments [1, 2]. These challenges are particularly pronounced for drugs targeting the central nervous system (CNS), where the restrictive permeability of the blood‐brain barrier (BBB) often decouples plasma drug levels from those in the brain. Consequently, measurements obtained from plasma or whole‐brain homogenates frequently fail to accurately reflect localized intracerebral drug levels, underscoring the critical need for direct monitoring of drug kinetics at specific brain regions [2, 3]. An analytical approach capable of continuous, real‐time monitoring of drug concentrations at target sites would enable a more in‐depth understanding of CNS drugs’ pharmacokinetics (PK), pharmacodynamics, and toxicity, thereby advancing precise clinical pharmacotherapy [4].

Current analytical approaches, including chromatography, mass spectrometry, and immunochemical assays, require discrete sampling and complex workflows, which limit the possibility of real‐time and in situ analysis [5]. Cerebral microdialysis offers continuous sampling but lacks sufficient temporal resolution [6], whereas optical strategies such as fluorescent approaches provide second‐scale monitoring yet are constrained by limited tissue penetration depth [7].

Electrochemical measurement quantifies target substances by directly or indirectly detecting electron transfer associated with redox reactions at the electrode interface. Owing to its high spatiotemporal resolution, this approach allows the precise quantification of neurochemicals in vivo [8, 9, 10, 11, 12]. A major advantage of electrochemical sensing is its operational timescale, which is substantially faster than conventional PK measurements, making it uniquely suitable for capturing rapid biochemical dynamics in living systems [13, 14, 15]. Recent technological advances have established carbon fiber microelectrodes (CFMEs) as a robust microsensor substrate for chronic in vivo monitoring, as they can be implanted in the brain or subcutaneous tissues with minimal tissue disruption while maintaining long‐term recording stability [16, 17, 18]. CFMEs have been successfully applied to detect a wide range of endogenous analytes, including dopamine (DA), ascorbic acid (AA), glutamate (Glu), serotonin (5‐HT), and adenosine, lactate, nicotinamide adenine dinucleotide (NADH), and to monitor glucose homeostasis [19, 20, 21, 22, 23, 24, 25, 26, 27, 28, 29, 30]. In contrast, real‐time monitoring of exogenous therapeutic drugs directly within the living brain remains largely unexplored.

Here, using safinamide (SAF) as a representative CNS drug, we demonstrate that the electrochemical approach enables continuous, stable, and selective in vivo tracking of drug dynamics in the rodent brain. As a medication recently approved for the management of motor fluctuations in mid‐ to late‐stage Parkinson's disease (PD) [31], SAF serves as an ideal model drug for quantifying cerebral PK, given that its therapeutic action occurs predominantly confined to the CNS. By integrating real‐time SAF monitoring with simultaneous neuronal activity recordings using a microelectrode array (MEA), we further investigated SAF‐induced alterations of neural activity under physiological conditions. Our findings demonstrate that this simple yet powerful electrochemical strategy provides physiologically relevant PK information and uncovers dynamic drug actions in the functioning brain. Importantly, the use of unmodified CFMEs confers distinct practical advantages, including straightforward fabrication, highly reproducible electrochemical performance across electrodes and batches, and excellent stability that enables reliable continuous recordings. Together, these features establish unmodified CFMEs as a robust and accessible platform for real‐time neuropharmacological measurements in vivo.

## 2 Results and Discussion

### 2.1 Development and Validation of an Electrochemical Strategy for In Vivo Safinamide Monitoring

To date, electrochemical detection of SAF has been restricted to in vitro systems [32], and its real‐time tracking in the living brain has not been reported. Using a glassy carbon electrode, we first characterized the electrochemical behavior of SAF and identified a stable redox pair with an oxidation peak at +1.0 V and a reduction peak at −1.4 V vs. Ag/AgCl (Figure S1a). This process corresponds to a reversible two‐electron transfer involving the secondary amine group of SAF (Figure S1b) [32]. The well‐defined and robust electrochemical activity of SAF on carbon surfaces, along with the inherent miniaturization and biocompatibility of CFMEs, suggests their potential as in vivo microsensors for SAF detection.

**FIGURE 1:**
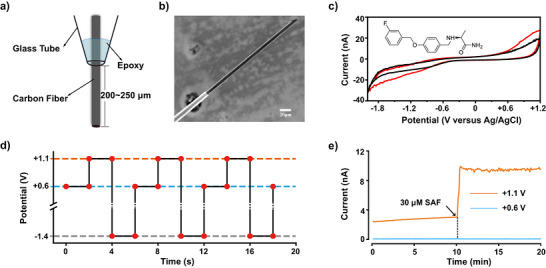
Electrochemical characterization of safinamide (SAF) at CFMEs and measurement setup. (a) Schematic diagram of the fabricated CFME. (b) Scan electron microscopy (SEM) image of the microelectrode tip. (c) Cyclic voltammograms (CV) recorded in 100 µM SAF in aCSF (red) and its corresponding control (black) at a scan rate of 0.1 V·s^−1^. The molecular structure of SAF is shown as an inset. (d) Schematic of the DPA waveform applied for SAF detection, illustrating the applied potentials and current sampling protocol as a function of time. (e) Representative DPA current responses to 30 µM SAF in aCSF. The applied parameters are: cleaning potential, −1.4 V (2 s); first pulse potential, +0.6 V (2 s); second pulse potential, +1.1 V (2 s).

CFMEs were fabricated by inserting a 7‐µm carbon fiber into a pulled glass pipette, leaving a protruding length of 200–250 µm for measurement (Figure 1a,b). In vitro cyclic voltammetry (CV) measurements using CFMEs in artificial cerebrospinal fluid (aCSF) containing 100 µM SAF revealed both oxidation and reduction characteristics of SAF (Figure 1c). Despite these well‐defined electrochemical responses, the relatively high oxidation potential of SAF (+1.0 V vs. Ag/AgCl) presents a substantial challenge for in vivo brain measurements. At such potential, endogenous electroactive species, including DA, AA, and norepinephrine (NE), which oxidize at much lower potentials (+0.2 V to +0.3 V) [23, 24, 33], can undergo co‐oxidation, resulting in severe interference for SAF detection. This fundamental selectivity challenge has largely precluded the real‐time tracking of SAF in the living brain to date.

Differential pulse amperometry (DPA), applying short voltage pulses and measuring the current difference before and after each pulse, provides improved analytical performance compared to classical amperometry, offering enhanced signal sensitivity and reduced susceptibility to background interference [34]. Previous studies have demonstrated that DPA can accurately reflect real‐time analyte concentrations while effectively suppressing background current interference [35]. Therefore, DPA was applied for SAF detection. In our DPA protocol for SAF measurement (Figure 1d), a cleaning potential of −1.4 V, corresponding to the reduction potential of SAF, was applied for 2 s without current sampling to minimize electrode fouling and refresh the surface. The potential was then stepped to +0.6 V for 2 s, below the oxidation threshold of SAF, followed by +1.1 V for 2 s, where SAF undergoes complete oxidation. The differential current, defined as Δ*I* = *I*_+ 1.1V_  − *I*_+ 0.6V_, was continuously recorded. In blank aCSF, the differential current remained stable, whereas the addition of 30 µM SAF produced a distinct stepwise increase in current at +1.1 V, while the +0.6 V signal remained unchanged as a background reference (Figure 1e). This differential measurement strategy effectively eliminated background and enabled precise quantification of SAF‐specific faradaic current.

**FIGURE 2:**
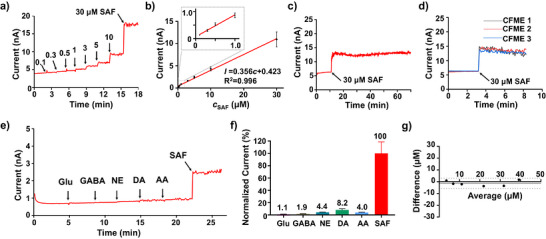
In vitro quantification of SAF using CFMEs. (a) Representative DPA current trace recorded during sequential additions of SAF (0.1–30 µM) into aCSF. (b) Calibration curve of steady‐state DPA current as a function of SAF concentration (mean ± SEM, n = 3). The inset shows the response at low SAF concentrations (0.1–1 µM). (c) Signal stability assessed by continuous monitoring of 30 µM SAF over 70 min. (d) Reproducibility of the current response to SAF across independently fabricated CFMEs (n = 3). (e) DPA current responses to potential interferents at physiological concentrations (Glu, GABA, NE, DA, 10 µM; AA, 100 µM) and SAF (10 µM). (f) Normalized current responses of interferents relative to that of 10 µM SAF (mean ± SEM, n = 3). (g) Bland–Altman analysis comparing spiked and measured SAF concentrations (spiked minus measured) vs. their average in rat cerebrospinal fluid. The solid gray line indicates the mean bias (−1.4 µM), and the dashed gray lines represent the 95% limits of agreement (± 1.96 SD; −5.8 to 3.0 µM).

We next validated the practical feasibility of the DPA protocol for SAF detection using CFMEs in vitro. Real‐time current signals were recorded as increasing concentrations of SAF were sequentially added into aCSF solution. As shown in Figure 2a, the differential current increased rapidly following each addition and reached a steady state within ∼6 s, a temporal resolution well‐suited for monitoring drug dynamics that typically evolves over minutes to hours in vivo. The steady‐state current exhibited a strong linear correlation with SAF concentrations ranging from 0.1 to 30 µM, yielding a limit of detection (LOD) of 95 nM (Figure 2b).

Moreover, the steady‐state DPA current remained within 92%–110% of its initial value (12.7 nA) during continuous monitoring of 30 µM SAF over 70 min (Figure 2c), indicating a highly reversible and reproducible oxidation process with no significant electrode passivation. This robust signal stability confirms the suitability of this DPA system for relatively long‐term in vivo recording of SAF. In addition, the electrochemical response was highly consistent across independently fabricated electrodes, with a relative standard deviation (RSD) of 7.2% for three CFMEs fabricated on different days (Figure 2d). This low inter‐electrode variability confirms the reliability of electrode surface properties and enables direct comparison across experiments.

Selectivity is a critical concern for in vivo measurements since the brain contains abundant species such as glutamate (Glu), γ‐aminobutyric acid (GABA), NE, DA, and AA [9, 16, 36, 37, 38, 39, 40]. Glu and GABA are shown to be electrochemically inactive, while other electroactive compounds possess relatively low oxidation potentials and are fully oxidized at +0.4 V (Figure S2). In the presence of physiological concentrations of potential interferents, the maximum signal change observed with DA was only ∼8.2% of that from 10 µM SAF (Figure 2e,f) under the optimized DPA conditions for SAF. This corresponds to an approximately 12‐fold signal lower response compared with 10 µM SAF, demonstrating that the method enables reliable in vivo SAF monitoring with minimal interference from endogenous species. Considering that the electrochemical signal of SAF arises from oxidation of its secondary amine, the potential interference from its metabolites is minimal. The major metabolite, NW‐1689, lacks this functional group, while the minor metabolite, NW‐1153, is present at low levels and is rapidly converted to NW‐1689. Therefore, their contribution to the measured signal is negligible [41, 42].

In comparison, at a constant applied potential of +1.1 V, NE, DA, and AA produced substantial interference in the direct *i‐t* measurements (Figure S3), indicating that the *i‐t* method fails to eliminate interference from endogenous electroactive species. These comparative results further highlight the advantages of the DPA method for SAF detection.

During in vivo measurements, endogenous macromolecules may adsorb onto the electrode surface, leading to its passivation and thus compromising its detection sensitivity and accuracy [43]. To assess sensor performance under biologically relevant conditions, we conducted standard‐addition experiments by spiking defined concentrations of SAF (4, 6, 10, 20, 30, and 40 µM) into undiluted rat cerebrospinal fluid (CSF) and measuring the corresponding current responses. Quantification accuracy in this concentration range was calibrated using an external calibration curve. As shown in Figure 2g, Bland–Altman analysis revealed a small bias of −1.4 ± 2.2 µM (mean ± SD of the differences between spiked and measured concentrations). All data points fell within the 95% confidence interval range of −5.8 to 3.0 µM. This minimal systematic deviation demonstrates that the DPA approach enables accurate and reliable quantification of SAF in physiologically relevant CSF environments, supporting their suitability for in vivo measurements in the brain.

### 2.2 Real‐Time In Vivo Pharmacokinetic Profiling of Safinamide in the Substantia Nigra

**FIGURE 3:**
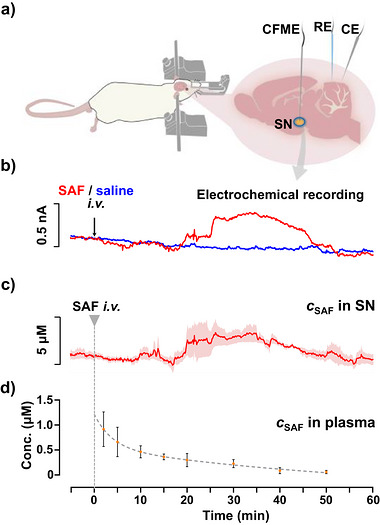
In vivo real‐time monitoring of SAF PK in the SN. (a) Schematic of the in vivo experimental setup, showing implantation of a CFME into the SN for SAF detection, with the reference (RE) and counter (CE) electrodes positioned on the dura. (b) Representative real‐time DPA current responses following intravenous administration of SAF (40 mg·kg^−1^, red) or saline control (blue). (c,d) Average SAF concentration‐time profiles measured in the SN (c) and in plasma (d). Plasma data were fitted with a biphasic decay model. Data represent mean ± SEM (n = 4).

Considering the excellent performance of this analytical approach, we next evaluate its capability for real‐time monitoring of SAF in the living rat brain. Under PD pathology, astrocytes surrounding dopaminergic neurons in the SN exhibit significantly elevated monoamine oxidase B (MAO‐B) expression. Pharmacological inhibition of MAO‐B by SAF restores DA levels and alleviates motor symptoms, making the SN a physiologically relevant target region for in vivo monitoring of SAF [44]. For these measurements, a CFME was stereotaxically implanted into the SN, while the reference (RE) and counter (CE) electrodes were positioned on the dura mater (Figure 3a). Histological verification via hematoxylin and eosin (HE) staining with Evans blue injection at the target coordinates confirmed correct tip placement in the SN (Figure S4).

Real‐time in vivo drug monitoring was conducted in four anesthetized rats. Following establishment of a stable baseline, during which the electrode equilibrates with the local brain microenvironment, thereby mitigating subsequent fouling effects on SAF measurements [45, 46, 47], SAF (40 mg·kg^−1^) was administered intravenously via the tail vein. This dose was chosen to achieve clinically relevant plasma exposure for direct PK characterization [48]. No comparable signal change was observed in control rats receiving a saline injection (Figure 3b). However, the injection of SAF produced a distinct increase in the DPA current, which reached a peak and subsequently declined toward baseline (Figure 3b). To verify the specificity to SAF, the current responses at +1.1 V and +0.6 V were analyzed separately (Figure S5). The current at +0.6 V remained stable throughout the recording period, with no significant deviation from the pre‐injection baseline following either saline or SAF administration. This stability indicates that brain matrix and electrode fouling show no significant influence on SAF measurement. In contrast, the current at +1.1 V showed no change after saline injection but increased significantly following SAF administration and gradually returned to baseline. Together, these results demonstrate that the recorded DPA signal is specific to SAF. On average, the peak current response (I_max_) reached 0.30 ± 0.07 nA, corresponding to an estimated maximum SAF concentration (C_max_) of 2.57 ± 0.98 µM (Figure 3c), with an area under the curve (AUC) of 60.9 ± 13.6 min·µM. Fitting the decay phase with a mono‐exponential decay model yielded an intracerebral elimination half‐life (t_1/2_) of 49.3 ± 6.73 min (Table S1).

To directly compare SAF's central and systemic PK, plasma samples were collected from the orbital sinus at defined time points following SAF injection. Plasma SAF concentrations were analyzed using a validated high‐performance liquid chromatography‐mass spectrometry (HPLC‐MS) method and fitted with a biphasic exponential decay model. Comparison of key PK parameters revealed substantially higher SAF exposure in the SN than in plasma, with C_max_ and AUC values approximately 2.8‐fold (2.57 ± 0.98 µM vs. 0.93 ± 0.17 µM) and 4.5‐fold (60.9 ± 13.6 vs. 13.5 ± 2.06 min·µM) greater, respectively (Figure 3c,d and Table S1). This pronounced brain enrichment is likely attributable to SAF's lipophilicity and high BBB permeability, coupled with its higher binding affinity for the extravascular tissue compared to plasma protein, ultimately leading to its elevated concentration in the CNS [48]. Therefore, this also demonstrates that conventional PK evaluation based solely on blood measurements provides an inaccurate assessment for drugs acting on the CNS.

Notably, the PK profiles of SAF in the SN and plasma exhibited significant temporal differences. Following intravenous administration, SAF was rapidly absorbed into the systemic circulation (T_Cmax_, 2.75 ± 0.75 min), after which plasma concentrations declined in a biphasic manner. By 20–30 min post‐injection, plasma SAF levels continued to decrease gradually (Figure 3d). In sharp contrast, SAF levels in the SN remained at a sustained plateau during 20–35 min post‐ injection window (Figure 3c), despite ongoing systemic clearance. Subsequent fitting of the data to exponential equations for one‐ and two‐compartment models (as described in the SI Methods) yielded a half‐life (t_1/2_) of 49.3 ± 6.73 min in the brain and 28.8 ± 1.70 min in plasma (Table S1). These results indicate that SAF exhibits strong affinity for brain tissue and relatively slow clearance, leading to prolonged intracerebral exposure. It is well established that the pharmacological effects of CNS drugs are determined by drug distribution at the brain target site rather than systemic levels [49, 50]. Accordingly, the prolonged retention of SAF in the SN may contribute to its sustained central pharmacological effects. Moreover, the continuous in vivo monitoring enabled by DPA allows accurate determination of individual PK parameters, capturing inter‐animal variability and providing a physiologically relevant, subject‐specific characterization of SAF's central PK profile.

### 2.3 In Situ Safinamide Accumulation in the Superior Colliculus and Its Impact on Vision‐Related Neuronal Activity

**FIGURE 4:**
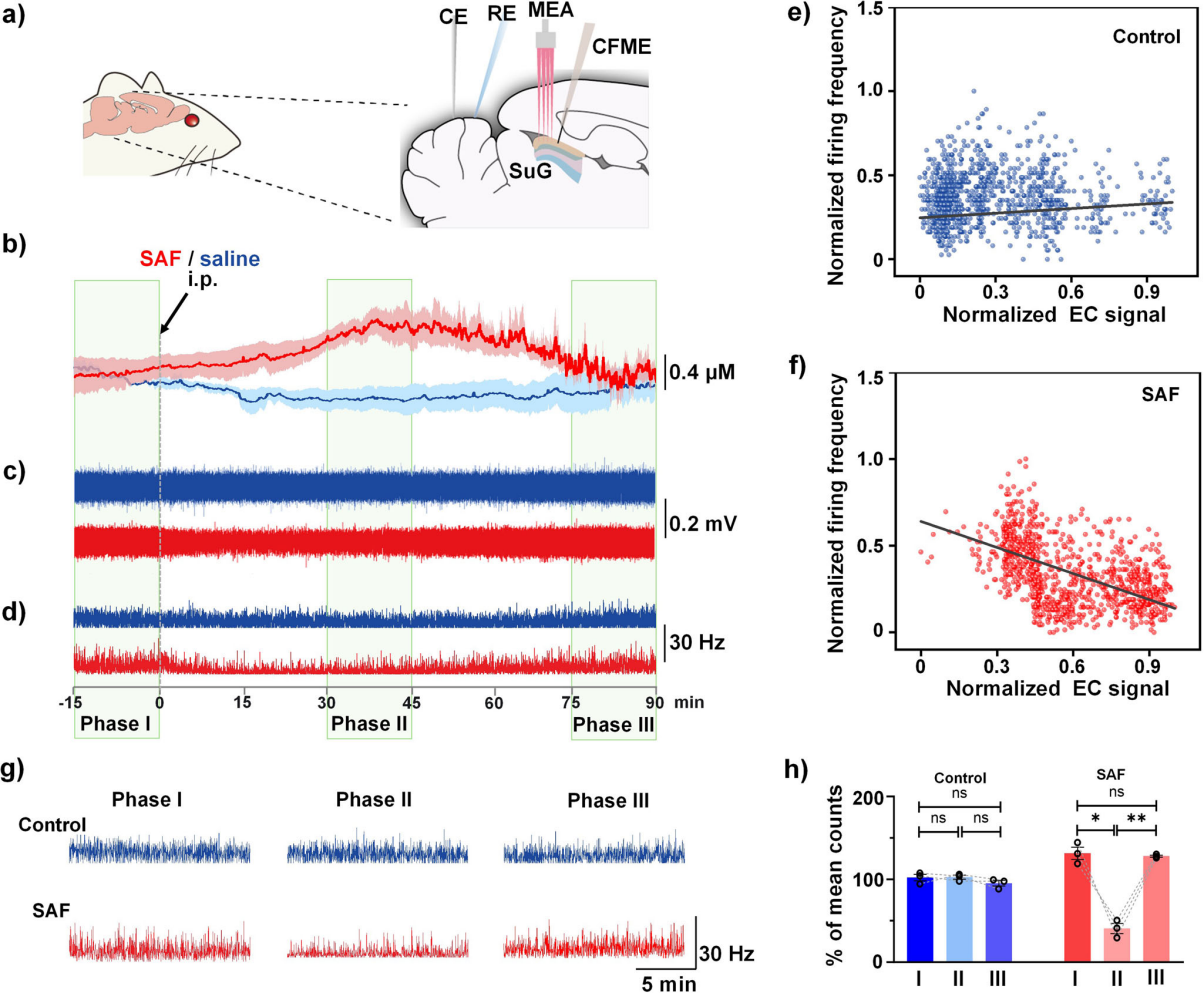
In vivo real‐time tracking of SAF concentration dynamics and associated neuronal activity in the SuG of the SC. (a) Schematic of the experimental setup, showing implantation of a CFME and a MEA into the SuG for simultaneous SAF monitoring and neuronal recording. The RE and CE electrodes were positioned on the dura. (b) Real‐time current responses following intraperitoneal administration of 80 mg·kg^−1^ SAF (red) or saline control (blue). Data are shown as mean ± SEM (solid line with shaded area, n = 6). (c,d) Representative spontaneous neuronal action potential (c) and corresponding firing frequencies (d) recorded by the MEA following administration of SAF (red) or saline control (blue). (e,f) Spearman correlation coefficient analysis of electrochemical (EC) signal and neuronal firing for saline control (e) and SAF‐treated group (f). (g) Expanded neuronal firing traces corresponding to the three time‐windows (phases I–III) indicated in panels (c) and (d). (h) Comparison of normalized mean counts of neuronal firing across the three phases (n = 3). Statistical significance was evaluated using paired *t*‐tests. ^**^ *p* < 0.01; ^*^ *p* < 0.05; ns, *p* > 0.05. [Correction added on 18 May 2026, after first online publication: figure 4 is updated in this version.]

Preclinical studies have demonstrated that high‐dose SAF induces retinal degeneration in rats, prompting recommendations for systematic ophthalmic monitoring in patients with pre‐existing retinal disorders such as albinism, uveitis, or inherited retinopathies [51]. However, the neural mechanisms underlying SAF‐induced visual impairment remain poorly understood. In rodents, approximately 90% of retinal ganglion cells (RGCs) project to the SuG of the SC, with each SC neuron receiving convergent inputs from approximately six RGCs. Extensive prior studies have documented both morphological and functional deficits of retinal neurons across diverse degeneration models [52], highlighting the SuG as a critical target for investigating drug‐induced effects in the visual pathway. Building on this anatomical and functional basis, we applied our established in vivo DPA platform to quantitatively monitor SAF concentration dynamics in the SuG and characterized drug‐induced modulation of visual pathway activity monitored simultaneously with in vivo electrophysiological recordings. As illustrated in Figure 4a, a CFME was implanted into the SuG of an anesthetized rat. Histological results confirmed that the tip was correctly positioned in the SuG (Figure S6). After stabilization of the baseline signal, SAF (80 mg·kg^−1^, a dose selected based on preclinical reports of SAF‐induced retinal degeneration in rats [48, 53, 54]) or saline was administered intraperitoneally, and intracranial current responses were continuously recorded. No comparable response was observed in the saline‐treated control rats. In contrast, SAF administration produced a clear electrochemical signal. Similar to SN, the current responses at +1.1 V and +0.6 V in the SuG confirmed the specificity of the recorded signal to SAF (Figure S7). The DPA current reached a mean peak time of 33.8 ± 4.2 min post‐injection and an average peak current of 0.38 ± 0.07 nA, which corresponds to a local SAF concentration of 0.69 ± 0.26 µM (Figure 4b and Table S1).

To examine the functional consequences of this local drug exposure, neuronal activity in the SuG was recorded under the same experimental conditions using an MEA (Figure 4c,d). The time of intraperitoneal injection was defined as time 0, with the intervals of −15 to 0 min (pre‐effect), 30–45 min (during effect), and 75–90 min (after effect) defined as phase I, phase II, and phase III, respectively. Baseline stability was first assessed by calculating the coefficient of variation of firing frequency over the 15 min pre‐effect period, together with linear regression analysis. The coefficient of variation was 19% ± 5% in the saline group, and 15% ± 4% in the SAF‐treated group, and no significant temporal trend was detected (*p* > 0.05), confirming stable baseline conditions (Figure S8). We next performed correlation analysis between neuronal firing frequency and electrochemical (EC) signals. No significant correlation was observed in the saline group (Spearman, R = 0.05, *p* = 0.1) (Figure 4e), consistent with the absence of changes in neuronal firing following saline injection (Figure 4g,h). In contrast, a significant negative correlation was observed in the SAF‐treated group (R = −0.51, *p* < 0.0001) (Figure 4f), indicating that the attenuation of neuronal firing is closely associated with SAF exposure. Further analysis revealed that SAF induced an extremely pronounced suppression of neuronal activity during phase II, followed by complete recovery in phase III (Figure 4d,g,h). Notably, the temporal profile of neuronal inhibition closely paralleled the SAF concentration–time curve in the SuG, with firing rates progressively declining as SAF accumulated and returning to baseline as the drug was cleared. This tight correspondence suggested a mechanistic link between elevated intracerebral SAF exposure and previously reported effects on the retina.

**FIGURE 5:**
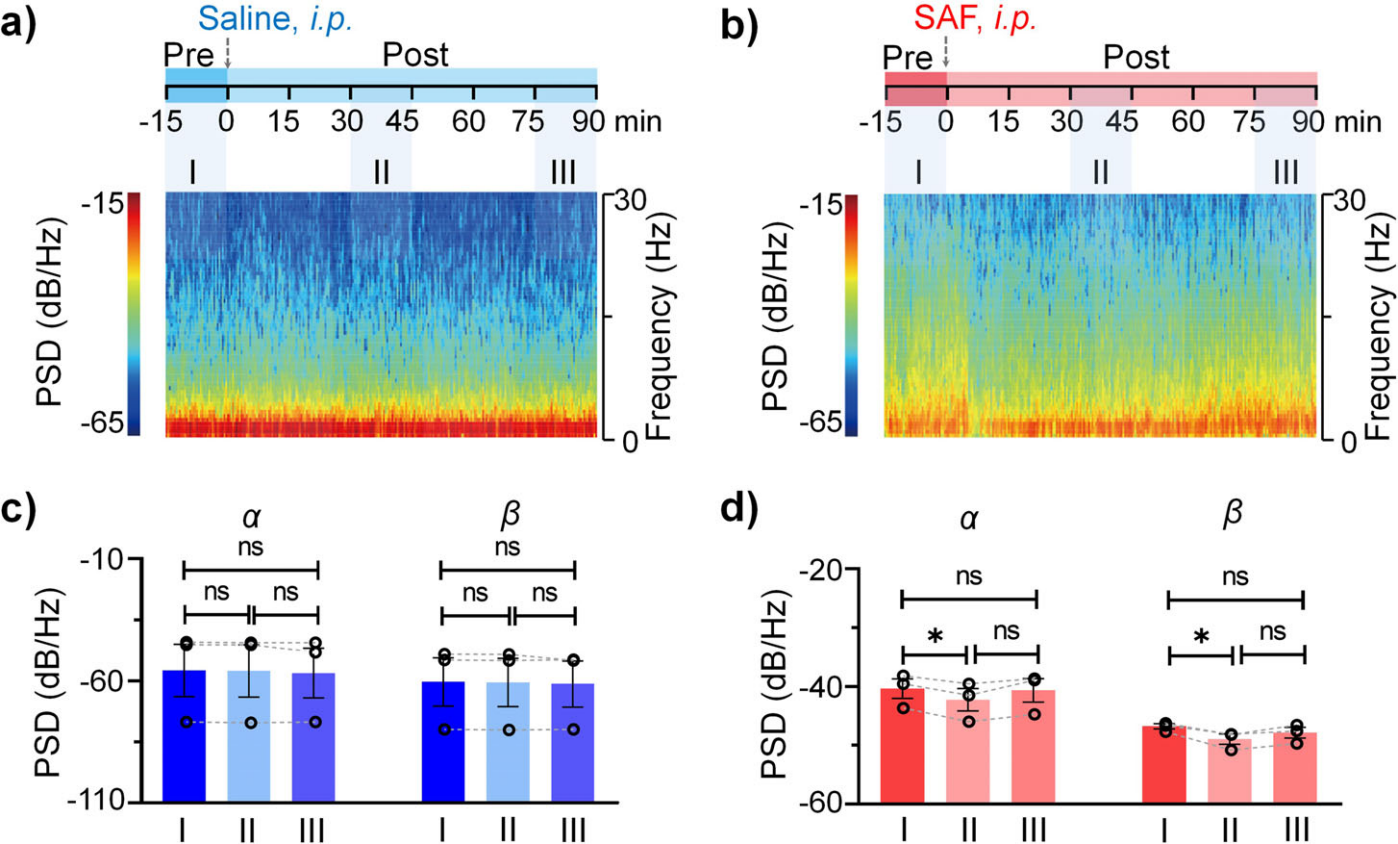
Modulation of SuG local field potential (LFP) by SAF. (a,b) Representative power spectral density (PSD) spectra of SuG LFPs recorded using MEAs from the control (a) and SAF‐treated (b) rats. Phases I, II, and III denote the pre‐effect, during effect, and after effect, respectively, each lasting 15 min. (c,d) Quantitative comparison of α‐ and β‐band across the three phases in control (c) and SAF‐treated (d) groups. Statistical significance was determined using paired *t*‐tests. ^*^ *p* < 0.05; ns, *p* > 0.05. [Correction added on 18 May 2026, after first online publication: figure 5 is updated in this version.]

To further quantify these effects at the population level, we subsequently analyzed local field potentials (LFPs) recorded from the SuG (Figure S9). Power spectral density (PSD) analysis [55, 56] revealed that low‐frequency oscillations (δ: 1–4 Hz and θ: 4–8 Hz) remained unchanged across all three phases in both SAF and control groups (Figures S10 and S11). In contrast, in the SAF group, PSDs of medium‐frequency α (8–13 Hz) and high‐frequency β (13–30 Hz) bands were significantly reduced during phase II compared to phase I and subsequently recovered in phase III, a pattern not observed in the saline controls (Figure 5). These frequency‐specific reductions indicate suppressed neuronal population excitability, potentially reflecting altered neural circuit activity or impaired synaptic transmission [57]. We speculate that this outcome may result from progressive accumulation of SAF in the SuG, where elevated concentrations inhibit neuronal activity.

**FIGURE 6:**
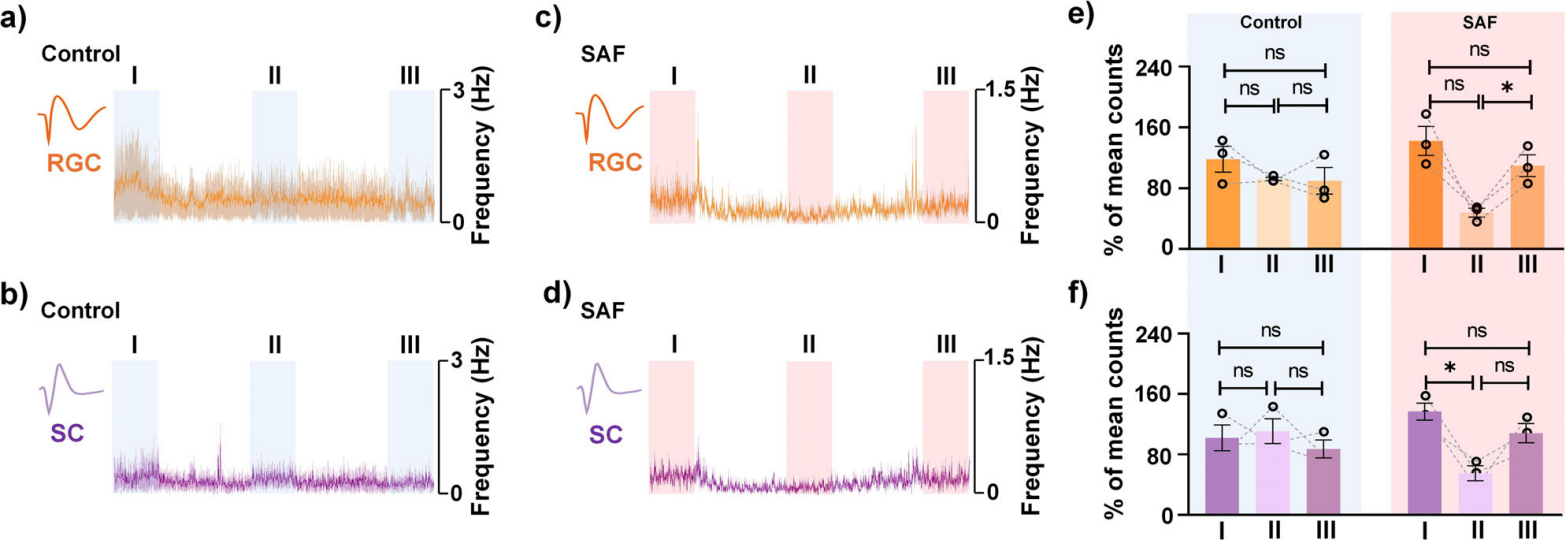
SAF‐induced modulation of neuronal firing frequencies in the SuG. (a–d) Neuronal firing frequencies of RGCs and SC neurons recorded using MEAs in the control (a,b) and SAF‐treated (c,d) groups. (e,f) Statistical comparison of relative mean counts of RGC (e) and SC (f) neurons across the three phases. Blue and pink shaded rectangles denote control and SAF‐treated groups, respectively. Data are shown as mean ± SEM (n = 3). Statistical significance was evaluated using paired *t*‐tests. ^*^ *p* < 0.05, ns, *p* > 0.05. [Correction added on 18 May 2026, after first online publication: figure 6 is updated in this version.]

RGCs and SC neurons, two main types existing in the SuG, can be discriminated by their distinct action potential waveforms. SC neurons exhibit biphasic spikes, whereas RGC neurons display triphasic waveforms arising from the combination of axonal and terminal potentials [58]. Leveraging this electrophysiological signature, we categorized recorded units and evaluated the effects of SAF on each neuronal population. Saline administration did not alter firing frequency or total spike counts across the three phases for either neuronal type (Figure 6). In contrast, increasing intracranial SAF concentrations were accompanied by graded reductions in firing frequencies for RGCs and SC neurons, with maximal suppression occurring during phase II (Figure 6c,d). Statistical comparisons demonstrated that total spike counts during phase II were significantly lower than those in both phase I and phase III for both neuronal populations, indicating a robust yet reversible inhibition of neuronal activity (Figure 6e,f, right). This reversibility agrees with the general principle that acute drug‐induced transient modulation of neuronal activity tends to resolve after discontinuation [59].

The SC serves as a central hub in the visual system. Anatomical and functional studies show that it not only receives direct retina input but also forms extensive connections with the thalamus, amygdala, basal ganglia, and cerebral cortex, thereby coordinating visual information processing and visually guided behaviors [60]. RGCs and SC neurons constitute tightly coupled elements of this circuitry, with RGCs providing the primary excitatory drive to SC neurons, reflecting their dense interconnectivity and functional synergy in maintaining the normal visual signal information [40, 58]. The comparable sensitivity of both neuronal populations to SAF suggests that elevated local SAF concentrations broadly suppress excitability across the retinocollicular pathway. Since the SC serves as a key relay linking the retina to multiple brain regions, suppression of its function may sequentially propagate to downstream visual‐related networks through its widespread projections [60, 61]. This suppression may arise from SAF‐mediated modulation of neuronal ion channels, such as Na^+^ or Ca^2+^ channels, leading to reduced membrane excitability, diminished action potential initiation, and ultimately weakened synaptic transmission [48, 57]. These effects provide a mechanistic basis for the alterations in visual function induced by SAF. Importantly, our findings imply that therapeutic strategies for SAF‐induced retinopathies should not be limited to local retinal interventions. Instead, greater attention should be paid toward the central components of the visual neuronal pathway, including the SC and other retinorecipient nuclei. Therefore, protecting the central nervous visual pathway may become a strategy to alleviate drug‐induced visual dysfunction.

## 3 Conclusion

In summary, we established a DPA‐based electrochemical platform for in vivo, real‐time tracking of drug concentration dynamics in the living brain. Using unmodified CFMEs, this approach achieves minimally invasive monitoring with high spatiotemporal resolution, excellent reproducibility, and robust performance in complex brain environments. The key challenge of selectively detecting SAF against endogenous interferents was addressed using DPA, which effectively suppressed background currents while amplifying drug‐specific faradaic signals. This strategy enabled stable, selective, and sensitive monitoring of SAF with micromolar sensitivity and second‐scale temporal resolution, allowing real‐time monitoring of intracerebral PK in individual animals, and comparing the heterogeneity of inter‐individual PK variability. By integrating electrochemical drug monitoring with neuronal electrophysiologic recordings, we further demonstrated that elevated SAF concentrations in visual brain regions temporally coincide with reversible suppression of neuronal activity, providing mechanistic insight into SAF‐associated effects on the retina reported in preclinical studies. Although currently limited to drugs with suitable redox properties, this study demonstrates the strong potential of in vivo electrochemical sensing as a powerful approach for directly probing drug PK and their effects on neuronal activity at their sites of action. Furthermore, although differences in drug dosing between brain regions limit direct quantitative comparison of the data, their qualitative complementarity provides a valuable analytical perspective for subsequent drug evaluation. Overall, this platform offers a practical and broadly applicable framework for advancing mechanistic neuropharmacology, improving the evaluation of CNS drug safety and efficacy, and ultimately enabling more rational and precise clinical drug use.

## 4 Methods

### 4.1 Reagents and Solutions

SAF was obtained from Beijing Beite Renkang Biomedical Technology Co., Ltd. Heparin sodium solution (0.1%, 125 U/mL) was obtained from Leagene. Isoflurane was purchased from Beijing Keye Xingcheng Technology Co. Other chemical reagents, of analytical grade, were purchased from Sigma–Aldrich and used as obtained. The aCSF was prepared by mixing NaCl (126 mM), KCl (2.4 mM), KH_2_PO_4_ (0.5 mM), MgCl_2_ (0.85 mM), NaHCO_3_ (27.5 mM), Na_2_SO_4_ (0.5 mM), and CaCl_2_ (1.1 mM) into Milli‐Q water, and the solution pH was adjusted to 7.4. All aqueous solutions were prepared with Milli‐Q water. Unless expressly stated otherwise, all experiments were carried out at room temperature.

### 4.2 Fabrication of Carbon Fiber Microelectrodes

CFME (7 µm in diameter) was fabricated following a previously described method [16]. Briefly, a single carbon fiber was inserted into a glass capillary tube (outer diameter 1.5 mm, inner diameter 0.84 mm). The capillary was then pulled into two separate capillaries using a microelectrode puller (PC‐100, Narishige, Japan). The exposed carbon fiber at the tip of the glass capillary was trimmed to a length of 200–250 µm under a microscope using a surgical scalpel. The electrode tip was subsequently immersed in epoxy solution for 2 min to seal, followed by a ∼10 s acetone soak. The electrode was then cured at 100°C for 1 h. Finally, conductive silver glue was applied to securely attach a conductive copper wire to the carbon fiber inside the glass capillary.

### 4.3 In Vitro Electrochemical Measurements of Safinamide

All electrochemical measurements, including CV and DPA, were performed using a computer‐controlled electrochemical analyzer (CHI 760e, Shanghai, China) in a standard three‐electrode cell setup at room temperature. For CV electrochemical measurements, a glassy carbon electrode (3 mm in diameter) or a CFME was used as the working electrode. The working electrode, CE, and RE were immersed in an electrochemical cell containing aCSF buffer solution. CV measurements were performed in 10 mL of aCSF solution in the presence and absence of 100 µM SAF. In the case of the glassy carbon electrode, it was polished with 0.5 and 0.05 µm alumina slurry on a polishing cloth, followed by ultrasonic rinsing with ethanol and Milli‐Q water for 2 min each. The electrode was then thoroughly rinsed with Milli‐Q water and dried with nitrogen gas before use to ensure a clean and mirror‐like surface prior to each measurement [16].

For the electrochemical measurement of DPA, the electrochemical properties of SAF were characterized using a CFME system. The three‐electrode system was transferred to the aCSF buffer solution, and a stock solution of SAF at the desired concentration was added to the buffer solution. Step potentials were sequentially applied to +0.6, +1.1, and −1.4 V, with each potential held for 2 s. The difference in anodic currents recorded at +0.6 and +1.1 V was measured. And the solution was stirred using a magnetic stir bar during the measurements. The current difference was plotted as a function of SAF concentration in aCSF to construct a calibration curve, which can be used for in vitro or in vivo calibration of CFMEs for SAF detection. The same measurement procedure was applied for the detection of SAF in biological samples.

### 4.4 In Vivo Measurements of Safinamide in the Brain

Adult male Sprague−Dawley rats weighing between 200 and 300 g were purchased from Peking University Health Science Center (Beijing, China). The animals were housed under a 12 h light/12 h dark cycle with free access to food and water. All animal use and care procedures in the in vivo experiments were conducted in accordance with the guidelines approved by the Institutional Animal Care and Use Committee (IACUC) of Peking University (Accreditation number: BCJH0232).

The in vivo animal experiments were performed as described previously [62]. Briefly, during the in vivo experiments, the animals were anaesthetized with isoflurane (4% for induction, 2% for maintenance) through a gas pump (RWD R520, Shenzhen, China) and secured onto a stereotaxic frame. A prepared CFME was carefully implanted into the right SN (coordinates: anteroposterior, −5.5 mm; mediolateral, −2.2 mm; depth, 8.3 mm) or the SuG (coordinates: anteroposterior, −6.5 mm; mediolateral, −1.5 mm; depth, 3.5 mm) of the rat using standard stereotaxic procedures. The RE and CE were positioned into the dura of the brain. The electrode system was then connected to the electrochemical workstation, and the same DPA procedure used for in vitro measurements was applied. After the background signal stabilized, drugs were administered, and real‐time current data were plotted and analyzed.

The 16‐channel gold‐based MEA was purchased from the Institute of Semiconductors, Chinese Academy of Sciences (Beijing, China). A 30 µm‐thick 4 × 4 array (probe spacing: 125 µm; probe length: 10 mm) with 16 recording sites (diameter: 20 µm) was prepared. The spacing distance between two adjacent electrode sites was 100 µm. To record the electrical activities of neurons, an MEA was implanted in the rat SuG brain region for electrophysiology recording. Three small screws were fixed into the rat skull with attachment to the brain fluid, and a silver wire was connected to the screw to ground the brain fluid with the OmniPlex‐128D instrument (Plexon Inc., Dallas, Texas, USA) [8, 23]. During our experiments, spontaneous spike firing signals were sampled at a rate of 25 kHz with a high‐pass filter applied at 250 Hz to view the neural spikes on the OmniPlex‐128D instrument and recorded before and after saline or SAF injection. All recorded single‐unit spikes were analyzed in the Plexon Offline Sorter by automated t‐distribution expectation maximization and manual clustering to sort out different neurons, spike histogram (6 s bins) for each sorted neuron, and spectrograms were generated by the Plexon NeuroExplorer to examine possible changes in firing frequencies induced by saline or SAF. Throughout the entire surgical procedure, the animal's body temperature was maintained at 37°C using a heating pad.

### 4.5 Brain Tissue Hematoxylin and Eosin Staining

To assess targeting accuracy, ex vivo histology was performed following the completion of in vivo experiments. The rats were first transcardially perfused with 0.9% saline, followed by 4% paraformaldehyde to fix the brain tissue. After perfusion, the brains were carefully removed and dehydrated through a graded series of ethanol solutions. The dehydrated tissues were then embedded in paraffin wax using standard embedding molds. Coronal sections of the brain were cut at a thickness of 20 µm using a microtome. After sectioning, the slides were dried and subsequently subjected to HE staining. The slides were first stained with hematoxylin solution for 5–10 min, followed by rinsing under water for 10 min to remove excess stain, and then briefly washed with distilled water for a few seconds. Differentiation was carried out for 2–30 s, after which the samples were again rinsed under water for 10 min. Subsequently, the samples were stained with eosin solution for 30 s to 2 min. Finally, the samples were dehydrated through a graded ethanol series, cleared in xylene for 5 min, and mounted for microscopic examination.

### 4.6 Data Analysis

Prior to in vivo experiments, the LOD of the CFME was determined in vitro. The background current was recorded for 30 s in pure aCSF solution. The LOD was then calculated using the SD of this background current and the slope of the calibration curve, as described in Equation (1) [1]:

(1) $$\begin{array}{c} LOD=\frac{3SD}{slope}\;\# \end{array}$$

The concentration‐time data of SAF in the brain were fitted to a monophasic exponential decay model (Equation 2), while the concentration‐time data of SAF in plasma were fitted to a biphasic exponential decay model (Equation 3) [1]:

(2) $$\begin{array}{c} C=Ae^{-\;\alpha t}\# \end{array}$$

(3) $$\begin{array}{c} C=Ae^{-\alpha t}+Be^{-\beta t}\;\# \end{array}$$

where C is the drug concentration, A and B represent mathematical coefficients (the maximum concentrations corresponding to the drug distribution phase and elimination phase, respectively), α and β are the elimination rate constants (i.e., the slopes of the rapid and slow elimination phases of drug disposition, respectively), and t is time. C_max_ and T_Cmax_ were directly obtained from the experimental raw data of the SAF concentration–time curve, and the local brain t_1/2_ and plasma t_1/2_ were derived from the α value in Equation (2) and the β value in Equation (3), respectively. The AUC was calculated using the definite integral of the SAF concentration–time curve ranging from drug injection to when the drug concentration reached zero, as summarized in Table S1.

### 4.7 Statistical Analysis

No outlier exclusion was performed unless explicitly noted in the figure legends. The sample size (n) for each analysis corresponds to the number of independent biological replicates or animals, as indicated in the corresponding figure legends. Statistical comparisons between two paired groups were conducted using two‐tailed paired t‐tests where applicable. The significance threshold was set at *p* = 0.05, and a *p*‑value of less than 0.05 was considered statistically significant. All statistical analyses were performed by GraphPad Prism 9.5.

## Conflicts of Interest

The authors declare no conflicts of interest.

## Supporting information




**Supporting File**: advs75618‐sup‐0001‐SuppMat.docx.

## Data Availability

The data that support the findings of this study are available on request from the corresponding author. The data are not publicly available due to privacy or ethical restrictions.
