# Supplementary material for: In Vivo Electrochemical Monitoring of Safinamide Pharmacokinetics in the Brain Explores Its Correlation With Vision‐Related Neuronal Activity
Source: Adv Sci (Weinh). 2026 May 10;13(43):e75618. doi: 10.1002/advs.75618 (PMC13335823; doi:10.1002/advs.75618)
Supplement: Supplementary file 1 — Supporting File: advs75618‐sup‐0001‐SuppMat.docx. [file ADVS-13-e75618-s001.docx]

**Supporting Information**

In Vivo Electrochemical Monitoring Safinamide Pharmacokinetics in Brain Explores Its Correlation with Vision-Related Neuronal Activity

Xiaoke Nan,^#[a]^ Chuqi Li, ^#[a]^ Qianhe Dai,^[a]^ Junlan Zhou,^[a]^ Lijiao Cao,^[a]^ Yuying Liu,^[a]^ Jinger Chen,^[a]^ Meiping Xiong,^[a]^ Yuhang Jiang,^[a]^ and Xianchan Li*^[a]^

[a] X. Nan, C. Li, Q. Dai, J. Zhou, L. Cao, Y. Liu, J. Chen, M. Xiong, Y. Jiang, Prof. X. Li
State Key Laboratory of Natural and Biomimetic Drugs, School of Pharmaceutical Sciences, Beijing Key Laboratory of Carbohydrate Intelligent Manufacture and Functional Applications
Peking University
Beijing 100191, P. R. China
E-mail: xcli@pku.edu.cn

^#^These authors contribute equally.

Contents

[Supplementary Figures and Tables S2](#_Toc226580519)

[Figure S1. Electrochemical characterization of SAF using a glassy carbon electrode. S2](#_Toc226580520)

[Figure S2. Electrochemical responses of endogenous interferents at the electrodes. S3](#_Toc226580521)

[Figure S3. Performance comparison of amperometry (+1.1 V) for SAF detection.. S4](#_Toc226580522)

[Figure S4. Histological verification of electrode placement in SN S5](#_Toc226580523)

[Figure S5. In vivo current responses recorded at +1.1 V and +0.6 V in SN. S6](#_Toc226580526)

[Figure S6. Histological verification of electrode placement in SuG S7](#_Toc226580527)

[Figure S7. In vivo current responses recorded at +1.1 V and +0.6 V in SuG . S8](#_Toc226580530)

[Figure S8. Baseline stability analysis of neuronal firing before saline or SAF injection. S9](#_Toc226580531)

[Figure S9. Spontaneous neuronal action potential recorded by the MEAs. S10](#_Toc226580532)

[Figure S10. PSD analysis in the control group. S11](#_Toc226580533)

[Figure S11. PSD analysis in the SAF-treated group. S12](#_Toc226580534)

[Table S1. Comparison of PK parameters of SAF in brain and plasma S13](#_Toc226580535)

# Supplementary Figures and Tables


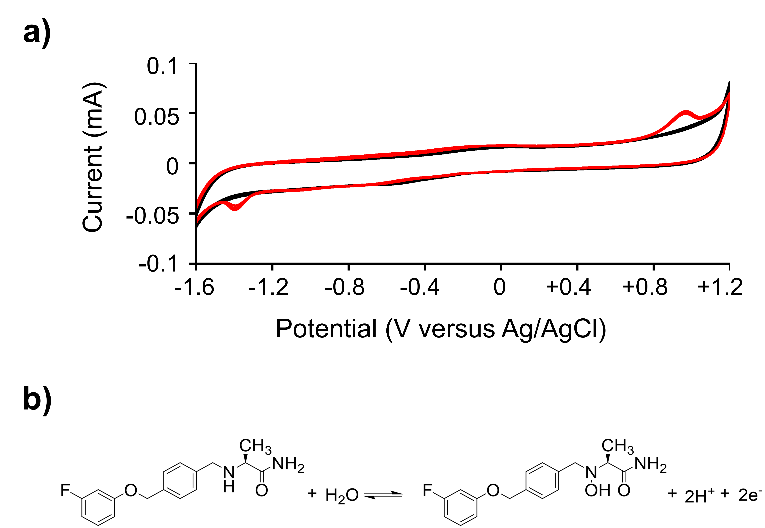


**Figure S1. Electrochemical characterization of SAF using a** **glassy carbon electrode**. **(a)** Cyclic voltammograms (CV) recorded in aCSF (black) and in the presence of 100 μM SAF (red) at a scan rate of 0.10 V·s^-^¹ for 5 consecutive cycles. **(b)** Schematic of the proposed redox mechanism of SAF at electrodes.


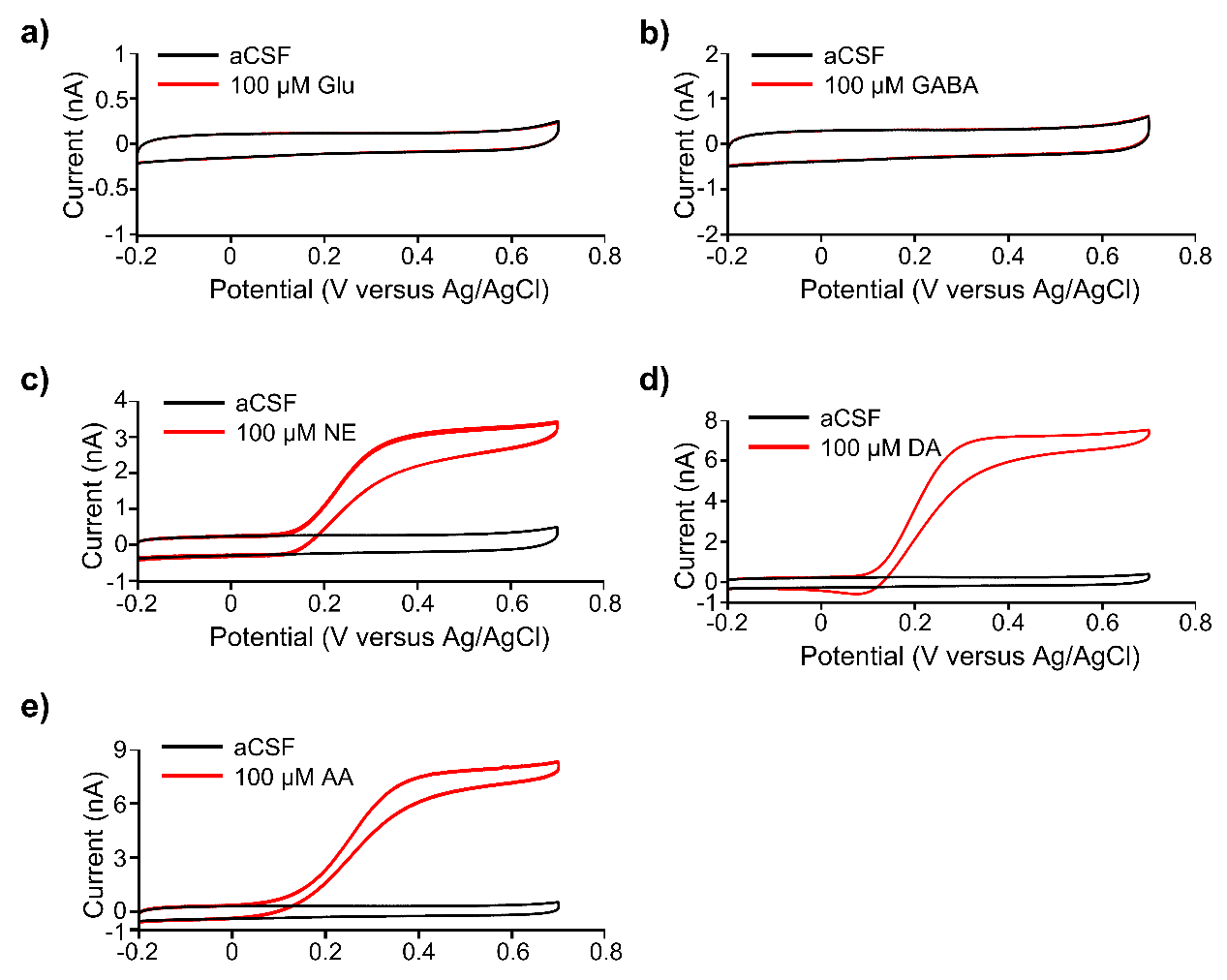


**Figure S2. Electrochemical responses of endogenous interferents at the electrodes**. CVs recorded at the CFMEs in aCSF (black) and in the presence of 100 μM Glu, GABA, NE, DA and AA (red) at a scan rate of 0.10 V·s^-^¹.

**Figure S3.** **Amperometry (*i-t*) performance for SAF detection**. **(a)** Amperometric current responses to potential interferents at physiological concentrations (Glu, GABA, NE, DA: 10 μM; AA, 100 μM) and SAF (10 μM) at +1.1 V (vs. Ag/AgCl). **(b)** Normalized current responses of interferents relative to 10 μM SAF (mean ± SEM, n=3). **(c)** Representative current trace during sequential additions of SAF (0.1–30 µM) in aCSF using *i-t* recoding at +1.1 V. **(d)** Calibration curve of steady-state current as a function of SAF concentration. Data are presented as mean ± SEM, n=3. Inset shows the response at low SAF concentrations (0.1-1 µM).

**Figure S4.** **Histological verification of electrode placement using HE staining in combination with Evans blue dye** **marking. (a)** A coronal brain section showing the SN. **(b)** Enlarged view of the red dashed box in **(a)**, where the arrow indicates the placed site of the CFME electrode.

**Figure S5. In vivo current responses recorded at +1.1 V and +0.6 V in the rat SN. (a, c)** Current-time (i-t) traces recorded at +1.1 V (orange) and +0.6 V (blue) following injection of saline **(a)** or SAF **(c)** in DPA measurement (mean ± SEM, solid lines with shaded areas). Sampling rate, 10 Hz. **(b, d)** Statistical comparison of current difference (post-injection minus pre-injection) following injection of saline **(b)** or SAF **(d).** Post-injection values were compared to pre-injection baseline using a paired *t*-test. Data are presented as mean ± SEM (saline, n=3; SAF, n=4). **p* < 0.05; ***p* < 0.01; ns, *p* > 0.05.

**Figure S6. Histological verification of electrode placement using HE staining in combination with Evans blue dye marking. (a)** A coronal brain section showing the SuG. **(b)** Enlarged view of the red dashed box in **(a)**, where the arrow indicates the placed site of the CFME and MEA electrodes.

**Figure S7.** **In vivo current responses recorded at +1.1 V and +0.6 V in the rat SuG. (a, c)** Current traces recorded at +1.1 V (orange) and +0.6 V (blue) following injection of saline **(a)** or SAF **(c)** in DPA measurement (mean ± SEM, solid lines with shaded areas). **(b, d)** Statistical comparison of current difference (post-injection minus pre-injection) following injection of saline **(b)** or SAF **(d).** Post-injection values were compared to pre-injection baseline using a paired *t*-test. Data are presented as mean ± SEM (saline, n=3; SAF, n=6). **p* < 0.05; ***p* < 0.01; ns, *p* > 0.05.

**Figure S8.** **Baseline stability analysis of neuronal firing in SuG before saline or SAF injection.** Linear regression of normalized firing frequency over the 15 min baseline period for saline **(a,** n=3**)** and SAF-treated **(****b,** n=3**)** groups. Dotted lines show linear regression fits. All slopes were not significantly different from zero (ns, *p* > 0.05).

**Figure S9. Representative spontaneous neuronal action potential recorded by the MEAs at the pre-effect (phase I, -15 to 0 min), during effect (phase II, 30-45 min), and after effect (phase III, 75-90 min) stages.** The black arrow marks the injection time (0 min).

**Figure S10. Power spectral density (PSD) analysis in the control group. (a)** Averaged PSD profiles across phases I, II, and III phases in control rats (mean ± SEM, solid line with shaded area, n=3). **(b, c)** Statistical comparison of the δ **(b)** and θ **(c)** frequency bands across phases (n=3). Data are presented as mean ± SEM. No statistical significance was found by paired *t*-test (ns, *p* > 0.05).

**Figure S11. PSD analysis in the SAF-treated group. (a)** Averaged PSD profiles across phases I, II, and III in SAF-treated group (mean ± SEM, solid line with shaded area, n=3). **(b, c)** Statistical comparison of the δ **(b)** and θ **(c)** frequency bands across phases (n=3). Data are presented as mean ± SEM. No statistical significance was found by paired *t*-test (ns, *p* > 0.05).

**Table S1. Comparison of PK parameters of SAF in different regions of brain and plasma**

| PK parameter | SN  (mean ± SEM) | Plasma  (mean ± SEM) | SuG  (mean ± SEM) |
| --- | --- | --- | --- |
| C_max_ (µM) | 2.57±0.98 | 0.93±0.17 | 0.69±0.26 |
| T_Cmax_ (min) | 30.2±3.6 | 2.75±0.75 | 33.8±4.2 |
| AUC (min·μM) | 60.9±13.6 | 13.5±2.06 | 16.1±4.1 |
| t_1/2_ (min) | 49.3 ±6.73 | 28.8 ±1.70 | 61.6±9.68 |
